# Interlaboratory Coverage Test on Plant Food Bioactive Compounds and Their Metabolites by Mass Spectrometry-Based Untargeted Metabolomics

**DOI:** 10.3390/metabo8030046

**Published:** 2018-08-24

**Authors:** Ville Mikael Koistinen, Andreia Bento da Silva, László Abrankó, Dorrain Low, Rocio Garcia Villalba, Francisco Tomás Barberán, Rikard Landberg, Otto Savolainen, Inmaculada Alvarez-Acero, Sonia de Pascual-Teresa, Christof Van Poucke, Conceição Almeida, Lucie Petrásková, Kateřina Valentová, Stephanie Durand, Wiesław Wiczkowski, Dorota Szawara-Nowak, Raúl González-Domínguez, Rafael Llorach, Cristina Andrés-Lacueva, Anna-Marja Aura, Tuulikki Seppänen-Laakso, Kati Hanhineva, Claudine Manach, Maria Rosário Bronze

**Affiliations:** 1 Institute of Public Health and Clinical Nutrition, University of Eastern Finland, P.O. Box 1627, FI-70211 Kuopio, Finland; kati.hanhineva@uef.fi; 2 Instituto de Tecnologia Química e Biológica, Universidade Nova de Lisboa (ITQB NOVA), Av. da República, 2780-157 Oeiras, Portugal; abentosilva@itqb.unl.pt (A.B.d.S.); salmeida@itqb.unl.pt (C.A.); mrbronze@ff.ulisboa.pt (M.R.B.); 3 Alkalmazott Kemia Tsz, Elelmiszertudomanyi Kar, Szent István Egyetem, 29-43 Villanyi Street, 1118 Budapest, Hungary; Abranko.Laszlo@etk.szie.hu; 4 INRA, Human Nutrition Unit, Université Clermont Auvergne, F63000 Clermont-Ferrand, France; dorrain.low-yanwen@inra.fr (D.L.); stephanie.durand.2@inra.fr (S.D.); claudine.manach@inra.fr (C.M.); 5 Department of Food Science and Technology, CEBAS-CSIC, Campus Universitario de Espinardo, edf 25, 30100 Murcia, Spain; rgvillalba@cebas.csic.es (R.G.V.); fatomas@cebas.csic.es (F.T.B.); 6 Division of Food and Nutrition Science, Department of Biology and Biological Engineering, Chalmers University of Technology, SE-412 96 Gothenburg, Sweden; rikard.landberg@chalmers.se (R.L.); otto.savolainen@chalmers.se (O.S.); 7 Department of Metabolism and Nutrition, Institute of Food Science, Technology and Nutrition (ICTAN-CSIC), Jose Antonio Novais 10, 28040 Madrid, Spain; inmaculada.alvarez@ictan.csic.es (I.A.-A.); soniapt@ictan.csic.es (S.d.P.-T.); 8 Technology and Food Science Department, Flanders Research Institute for Agriculture Fisheries and Food (ILVO), Brusselsesteenweg 370, B-9090 Melle, Belgium; christof.vanpoucke@ilvo.vlaanderen.be; 9 Laboratory of Biotransformation, Institute of Microbiology of the CAS, Vídeňská 1083, CZ-142 20 Prague, Czechia; petraskova@biomed.cas.cz (L.P.); kata.valentova@email.cz (K.V.); 10 Institute of Animal Reproduction and Food Research of the Polish Academy of Sciences, Tuwima 10, 10-748 Olsztyn, Poland; w.wiczkowski@pan.olsztyn.pl (W.W.); d.szawara-nowak@pan.olsztyn.pl (D.S.-N.); 11 Department of Nutrition, Food Science and Gastronomy, Pharmacy Faculty, University of Barcelona, Av Joan XXIII, s/n, 08028 Barcelona, Spain; raul.gonzalez@ub.edu (R.G.-D.); rafallorach@ub.edu (R.L.); candres@ub.edu (C.A.-L.); 12 CIBER Fragilidad y Envejecimiento Saludable (CIBERfes), Instituto de Salud Carlos III, 08028 Barcelona, Spain; 13 VTT Technical Research Centre of Finland Ltd., P.O. Box 1000, FI-02044 VTT, Espoo, Finland; Anna-Marja.Aura@vtt.fi (A.-M.A.); Tuulikki.Seppanen-Laakso@vtt.fi (T.S.-L.); 14 Instituto de Biologia Experimental Tecnológica (iBET), Av. da República, 2780-157 Oeiras, Portugal; 15 Research Institute for Medicines (iMed.ULisboa), Faculty of Pharmacy, University of Lisbon (FFULisboa), Avenida Professor Gama Pinto, 1649-003 Lisbon, Portugal

**Keywords:** phytochemicals, mass spectrometry, method development

## Abstract

Bioactive compounds present in plant-based foods, and their metabolites derived from gut microbiota and endogenous metabolism, represent thousands of chemical structures of potential interest for human nutrition and health. State-of-the-art analytical methodologies, including untargeted metabolomics based on high-resolution mass spectrometry, are required for the profiling of these compounds in complex matrices, including plant food materials and biofluids. The aim of this project was to compare the analytical coverage of untargeted metabolomics methods independently developed and employed in various European platforms. In total, 56 chemical standards representing the most common classes of bioactive compounds spread over a wide chemical space were selected and analyzed by the participating platforms (*n* = 13) using their preferred untargeted method. The results were used to define analytical criteria for a successful analysis of plant food bioactives. Furthermore, they will serve as a basis for an optimized consensus method.

## 1. Introduction

Plant bioactive compounds, or phytochemicals, represent up to thousands of plant-synthetized chemicals that do not act as nutrients, but may have other biological activities in vivo after plant consumption. The amount and diversity of these compounds present in food highly depend on genetic and environmental factors of the raw material, as well as food processing. Such factors include the species or cultivar, soil and climate conditions, storage conditions, fermentation, and the food preparation process. When consumed, phytochemicals are absorbed and metabolized by host and gut microbial enzymes with between-subject differences. The complex mixture of the derived metabolites may additively or synergistically contribute to the bioactivities and may account for the consistently reported beneficial health effects of diets rich in plant foods. A better knowledge about the effects and mechanisms of action of phytochemicals in vivo is necessary in order to develop new products and encourage the consumption of diets that are known to be healthy. To facilitate that, a deeper knowledge about the chemical composition of the diet and the true exposure of consumers to phytochemicals and their metabolites is essential, e.g., by utilizing untargeted and targeted metabolomics. In agricultural science, food science and nutrition, metabolomics has been applied from farm to fork and from fork to biofluids [1], including applications in food agriculture [2], food authenticity [3] and nutritional metabolomics [4]. There has been great development in technologies that can be used for that purpose, and mass spectrometry (MS) has become an indispensable method in analyzing plant bioactives in a quantitative or qualitative manner from various matrices [5,6,7,8,9,10]. In particular, liquid chromatography coupled with mass spectrometry (LC–MS) provides high mass accuracy, dynamic range and sensitivity due to advances in the instrumentation during the past decade, such as the development of ion cyclotron resonance (ICR), Orbitrap and quadrupole time-of-flight (QTOF) mass analyzers. Gas chromatography (GC–MS) has a clear advantage over LC–MS in being highly reproducible regarding retention times and mass spectral fingerprints of compounds [11]; however, the requirement of derivatization to increase the volatility of most analytes limits the usability of this technology to mainly primary metabolites [10,12].

Several targeted methods of analysis have been developed for different phytochemical families. Ultra-high-performance liquid chromatography coupled with mass spectrometry (UHPLC–MS) is the prevailing method for the analysis of polyphenols (e.g., flavonoids, phenolic acids, and lignans) in complex food matrices and human biofluids [8,10,13,14,15]. The majority of flavonoid analyses have been carried out using a reversed-phase (RP) C_18_ column, but more recently, hydrophilic interaction chromatography (HILIC) has emerged as a complementary option for the analysis of glycosylated flavonoids and other polar compounds [10]. GC–MS used to be the most frequently utilized technique for the detection of plant sterols, but as with several other groups of plant bioactives, the advances in LC–MS have decreased its utilization in the analysis of these compounds [16,17]. In the analysis of carotenoids from biological matrices, HPLC–MS with a reversed-phase C_8_, C_18_ or C_30_ column is often the method of choice [18]. Phase II metabolites of plant bioactives, such as polyphenol glucuronides and sulfates, have been analyzed with LC–MS from cocoa [19] and tea [20] as well as phenylacetamide sulfates in rye [21]. Alkylresorcinols, phenolic lipids that among edible plants exist almost exclusively in the outer layers of wheat, rye, quinoa (even-numbered alkyl chains) and barley grains, have been analyzed with GC–MS and normal-phase LC–MS [22]. Different combinations of HPLC, CE, UV and MS have been applied for the analysis of betaines [23], which are hydrophilic compounds containing a quaternary ammonium group. All these targeted methods were optimized to quantify a limited number of compounds belonging to the same family; some attempts have recently been made to simultaneously quantify a higher number of metabolites [15,24].

For a more extensive coverage of phytochemicals and metabolites, several targeted methods would need to be combined, which results in high costs. Untargeted metabolomics is a more comprehensive approach based on the detection of as many metabolite features as possible in a single analysis. It offers the possibility to identify unexpected or still unknown metabolites and to study the metabolic profiles of plant food bioactive compounds in a holistic way. It has been successfully applied to study differences in metabolites between food varieties, the impact of food processing on the product, and to find novel biomarkers of food intake as well as to investigate the associations between food exposures, food constituents and health outcomes [25,26,27,28,29].

There is no standardized method for untargeted metabolomics. Because of the extraordinary diversity of chemicals present in foods and biofluids, it is unlikely that one universal method using any of the current technologies can ever cover all of them. Metabolomics platforms independently develop their own analytical methods, generally one per sample matrix type, and ensure that the compound families of utmost importance for their research purposes are covered. The identification of the detected compounds is a difficult and burdensome task in untargeted metabolomics. It is facilitated by in-house reference libraries that compile the chromatographic and spectral data acquired for dozens or hundreds of authentic standards with the same analytical method. Unfortunately, in-house reference databases have major limitations when used in or transposed for a different analytical method. Depending on the chromatographic column and elution conditions, the type of mass spectrometer, the ionization and detection parameters, the chemical coverage and the analytical data can differ substantially between two methods.

The COST Action FA1403 POSITIVe (https://www6.inra.fr/cost-positive) is a large scientific network with the overall aim of investigating the interindividual variation in absorption, distribution, metabolism and excretion (ADME), and bioavailability, as well as the biological response to consumption of plant food bioactives. The aim is further to identify the main determinants involved and to propose strategies for the stratification of populations according to their responsiveness to these bioactives. One of the specific objectives is to explore the usefulness of untargeted metabolomics to better assess the true exposure of individuals to plant food bioactives and their metabolites. Thus, in the current study, a multiplatform test was carried out within participating research groups and metabolomics platforms in the POSITIVe Action to assess and compare the analytical coverage of previously developed untargeted profiling methods using a set of 56 selected plant food bioactives and metabolites that represent the most common classes of bioactive compounds in plants over a wide chemical space. We used unoptimized methods for the current analysis in order to acquire a realistic perception of the analytical coverage of existing methods, receive information about the characteristics affecting the analytical coverage of the compounds, and to assess whether some compounds are more easily detectable than others. Each platform analyzed the standards from both solvent and urine, to assess the matrix effect on the detection and identification of the compounds. The results from this work were used to define recommendations for a maximal coverage of plant food bioactive compounds and will serve as a basis for the development of a consensus method with optimized analytical coverage of these compounds.

## 2. Results and Discussion

### 2.1. Chemical Space

**Figure 1:**
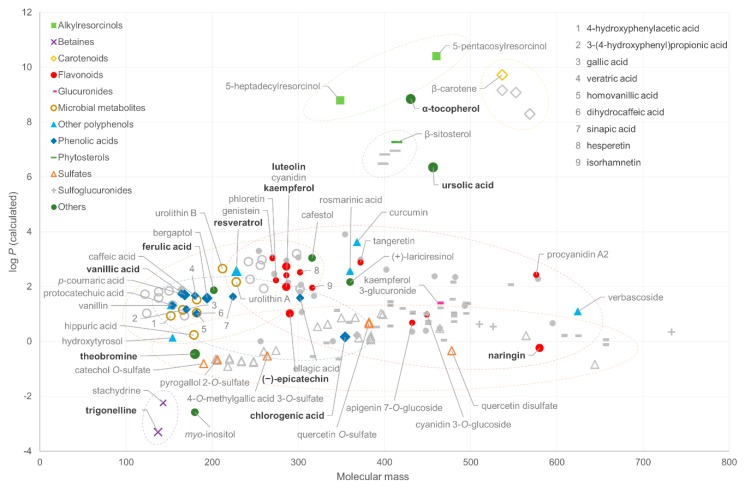
The chemical space (calculated log *P* as a function of monoisotopic molecular mass, MM) of the reference standards included in the analysis (colored markers), included in the initial list (including grey markers) and included in the proposed analytical coverage quality control mixture (highlighted compounds with bold text).

The chemical structures selected for the study were plotted based on their molecular mass and calculated log *P* value to visualize the chemical space of the phytochemical families represented (Figure 1). The masses of the compounds ranged from 122 to 733 Da, with betaines, phenolic acids and microbial metabolites having masses up to ~300 Da. Flavonoids are a chemically diverse group, ranging from relatively lipophilic aglycones, such as genistein, to more complex hydrophilic glycosides (naringin). Correspondingly, the addition of a hydrophilic quinic acid moiety in phenolic acids, such as chlorogenic acid, increases their hydrophilicity despite the increased mass. Sulfated and glucuronidated phytochemicals, which are often major metabolites in biological fluids, are spread over a wide mass range, because they are derivatives of both small phenolics and larger flavonoids, but all have a relatively low log *P* value. Sulfoglucuronides possess the highest mass because of their double derivatization. However, they could not be represented in the coverage test because of their poor commercial availability. Sulfate and glucuronide conjugates are also difficult to find commercially, and thus six compounds included in this study were synthetized in-house in the participating platforms (Table S1 in Supplementary Materials). Certain lipophilic families, including alkylresorcinols, carotenoids and phytosterols, and hydrophilic betaines, form very distinct groups in the plot due to their extreme log *P* values. The vast majority of the compounds are within the semi-polar range of the log *P* scale, and in further consideration of the mass range, they fall into the theoretical coverage of LC–MS platforms [30].

### 2.2. Detection of Compounds

**Figure 2:**
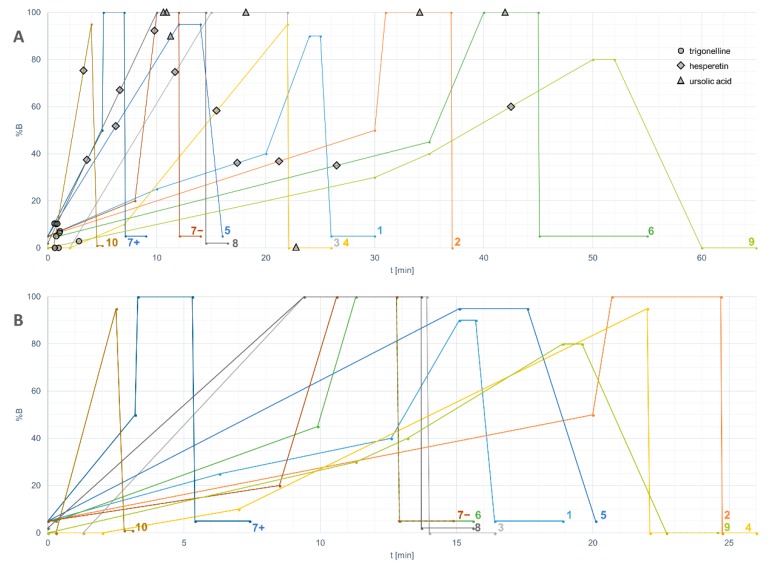
(**a**) HPLC gradients of the participating untargeted LC–MS platforms including the retention times of trigonelline, hesperetin and ursolic acid plotted against the percentage of solvent B. The numbers of the platforms correspond with Table 1. Platform 7 had separate HPLC methods for positive and negative ionization modes; (**b**) The same gradients normalized based on a column with dimensions of 2.1 × 100 mm, particle size 1.7 µm, and flow rate 0.4 mL/min.

Results were obtained from 13 platforms, including eight LC–QTOF platforms, one LC–TOF, one LC–QTRAP, one LC–QMS, and two GC–MS platforms (Table 1), reflecting the wide use of quadrupole time-of-flight LC–MS instruments in untargeted metabolomics studies. A variety of column types (*n* = 8) and lengths (ranging from 50 to 150 mm) was used in the LC–MS platforms. All platforms used a reversed-phase column based on C_18_ chemistry, and one platform used a complementary HILIC column. Acetonitrile was the choice of mobile phase in most platforms except platform 8, where methanol was used instead. 0.1–0.9% formic acid was frequently used as the acidic modifier to increase ionization of analytes in both positive and negative modes by all platforms except platform 7, which additionally included ammonium formate. The length and shape of the gradient, in which the proportion of the organic component of the mobile phase is changed gradually, varied greatly between the platforms even after the normalization (Figure 2).

**Figure 3:**
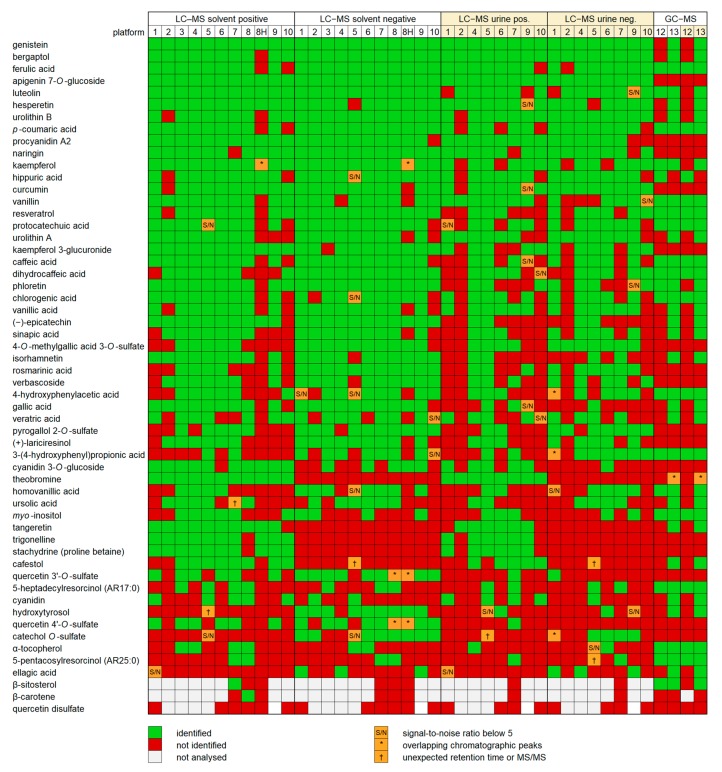
The positive identifications (green), undetected (red) and uncertain identifications (orange) of the chemical standards in different platforms, arranged in a descending order of the number of identifications in all analyses. Quercetin disulfate was not detected in any platform, suggesting that it was not present in the standard mixtures.

All platforms except the LC–QMS were capable of detecting and identifying the majority of the standards at least from the solvent mixture: the percentage of identifications ranged from 63% to 95% for the standard mixture in solvent and from 34% to 87% for the spiked urine. The LC–QMS instrument did not provide sufficient data to reliably identify any of the compounds, since a single quadrupole mass spectrometer is not well suited for metabolomics. For the other platforms, the results show that there is still room for optimization. The highest variability in the rate of detection was compound-specific; Figure 3 shows the identified compounds ranked in the order of positive identifications among both matrix types (solvent and urine samples) and ionization modes. Several flavonoids, such as genistein, bergaptol and luteolin, and some phenolic acids (ferulic and *p*-coumaric acid), were identified in nearly all platforms, ionization modes and matrices. Theobromine was only detected in the positive mode by LC–MS by all platforms and in both mixture types. Most semi-polar compounds, including the flavonoids and phenolic acids, were detected consistently by the LC–MS platforms. In contrast, highly lipophilic compounds, such as alkylresorcinols, carotenoids, phytosterols and α-tocopherol, and very hydrophilic compounds (betaines, sulfated flavonoids, *myo*-inositol and theobromine) were detected by fewer platforms, most likely due to their being outside of the polarity range of the column used or because of the chosen HPLC gradient being unsuitable for either end of the polarity range. Phloretin, which has been proposed as a biomarker of apple intake, was only detected in urine by half of the platforms. Among the most difficult compounds to detect was also ellagic acid, which has a very low solubility in common solvents (max 50 ppm) and thus may have been below the limit of detection in several platforms, as well as cyanidin, which has a low stability and may have deteriorated during the storage of some of the samples. Some of the platforms had difficulties in separating the isomers of quercetin sulfate, and quercetin disulfate was not detected by any platform, both possibly related to the relative lability of quercetin 4-*O*-sulfate and quercetin disulfate [31]. Certain compounds, such as epicatechin, gallic acid and isorhamnetin, were identified by nearly all platforms from the mixture in solvent but by less than half from the mixture in urine. In contrast, some typical metabolites found from urine, such as hippuric acid and urolithin A and B, were detected from the mixture in urine as often as from the mixture in solvent. Most compounds identified from the mixture in urine had a significantly lower signal-to-noise ratio compared to the mixture in solvent, suggesting that sensitivity issues caused by the matrix largely explain the absence of some identifications from the mixture in urine.

Depending on their chemical structure, some compounds can be detected in both positive and negative mode or only in one mode. Phenolic acids possess a carboxylic acid group in their structure, which is prone to deprotonation and results in a negative charge. However, most phenolic acids, apart from 3-(4-hydroxyphenyl)propionic acid, were also detected as less intense peaks in the positive mode in most platforms, possibly due to the use of formic acid, which enhances the protonation of the analytes. Sulfates are also more prone to ionization in the negative mode, and for flavonoids, the negative ionization is shown to provide higher sensitivity due to lower background noise compared to the positive mode [32]. Among all the analyzed standards, only catechol *O*-sulfate and ellagic acid were not detected in the positive mode in any of the platforms. In the negative mode, betaines (due to their inherent positive charge), cafestol, tangeretin and theobromine were not detected. In general, more detections were acquired in the positive mode, suggesting that in the case of only one ionization mode being applied, the positive mode might provide better coverage of plant bioactives in untargeted analyses. However, there may be bias towards certain compound classes or structures when using only one ionization mode, which detects certain types of compounds more easily, and the negative mode provides important complementary information, such as additional MS/MS spectra. In this study, the signal-to-noise ratio of phenolic acids and sulfates was generally higher in the negative mode, but flavonoids did not show consistently higher signal-to-noise ratios in the negative mode (data not shown).

The GC–MS platforms did not detect large (MM > 400) hydrophilic compounds, including flavonoid glycosides and sulfates, as these compounds suffer from low volatility even after TMS derivatization. Alkylresorcinols were detected more consistently in the GC–MS platforms compared to LC–MS. In general, based on the results in the current study, GC–MS is a suitable method for the untargeted analysis of secondary plant metabolites, but it does not offer major advantages regarding the analytical coverage when used as a complementary method with LC–MS. However, the high reproducibility of GC–MS and the availability of comprehensive spectral databases, such as NIST, may increase the identification rate. The low number of GC–MS platforms participating in this study limits the interpretation of their results. More advanced GC–MS instruments, such as two-dimensional gas chromatography coupled to time-of-flight MS (GC × GC–TOF–MS) could offer better peak separation and thus more coverage of compounds, although they would still be limited by the mass range.

### 2.3. Reliability of Identifications

**Figure 4:**
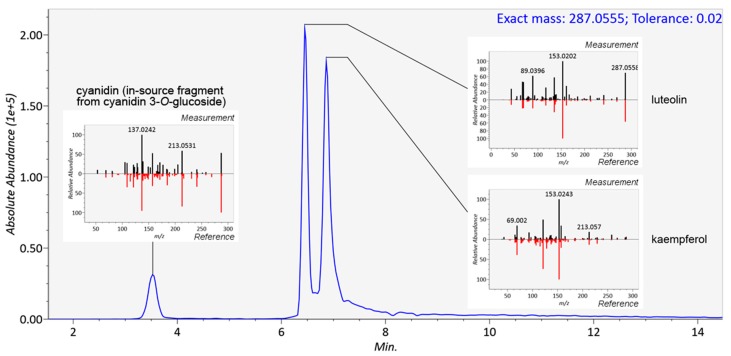
Separation and identification of three flavonoids, cyanidin, luteolin, and kaempferol, with the same molecular formula of the positive ion (C_15_H_11_O_6_^+^) in platform 8. The compounds were identified based on their MS/MS spectra, which were compared with reference spectra from publicly available databases [35]. Based on the retention time of cyanidin 3-*O*-glucoside, also present in the mixture, the feature appearing at RT 1.77 min in platform 7 and 3.5 min in platform 8 was identified as an in-source fragment (cyanidin ion).

**Figure 5:**
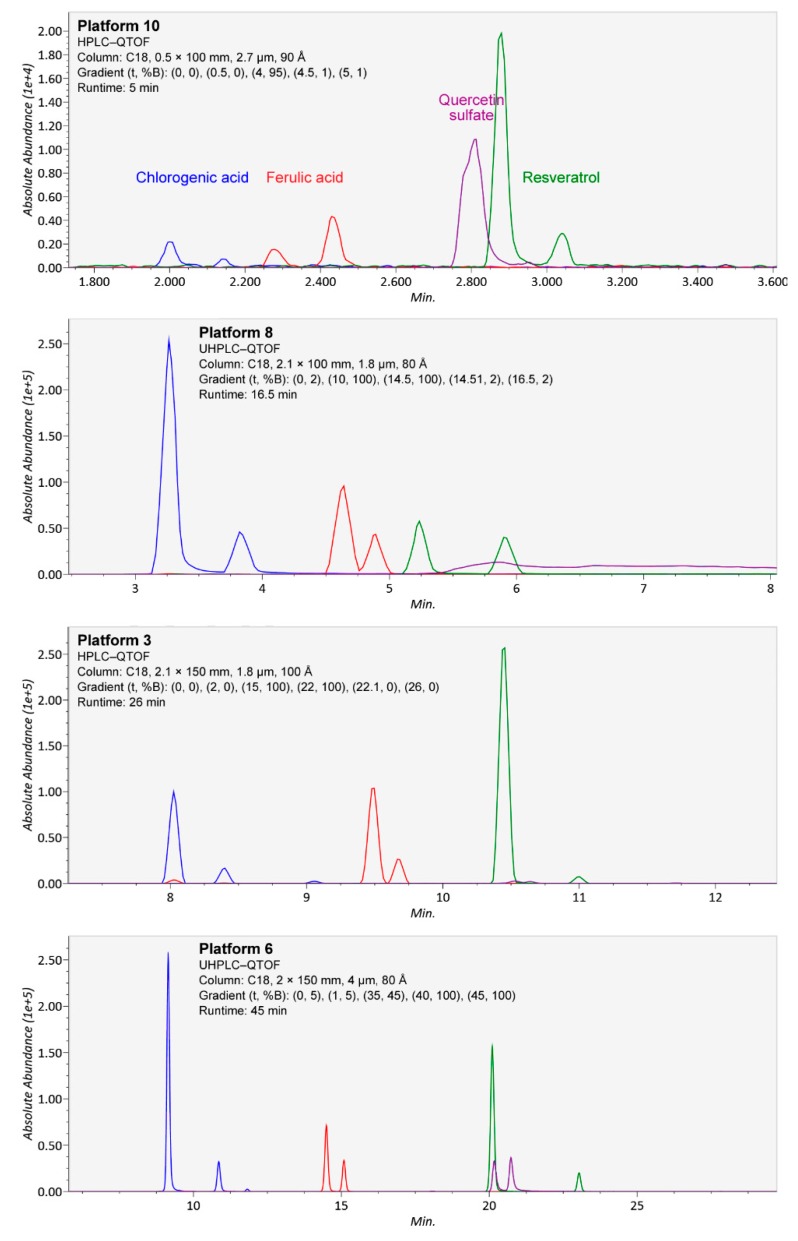
Separation of isomers from chlorogenic acid (blue), ferulic acid (red), resveratrol (green), and quercetin sulfate (purple; quercetin 3-*O*- and 4-*O*-sulfate, from separate chemical standards) in four platforms (10, 8, 3, and 6) using different chromatographic conditions, in ascending order of chromatography runtime. In the platforms, both forms of chlorogenic acid (3-*O*-caffeoylquinic and 5-*O*-caffeoylquinic acid) and the *cis* and *trans* isomers of ferulic acid and resveratrol, contained in single standards as mixtures, were well separated. However, in platforms 8 and 10, the isomers of quercetin sulfate were not separated due to tailing, and in platform 3, the compounds produced well-separated but weak signals.

In mass spectrometry, several measured parameters can be used complementarily for the identification of the analytes, such as retention time, elution order, exact mass, molecular formula generated from the isotopic pattern, and MS/MS fragmentation. In metabolomics, several levels of identification have been proposed, depending on the strength of evidence supporting the identification [33]. For a non-novel compound to be formally identified (level 1), a reference standard has to be analyzed in identical experimental conditions with two matching independent and orthogonal data, such as retention time and mass spectra. In untargeted metabolomics studies, reference standards are seldom available for all metabolites requiring identification, and therefore putative annotations (level 2) can be given based on, e.g., spectral matching with databases or earlier publications. Annotation based on exact mass only is not reliable, because several molecules from various chemical classes can share the same molecular formula (and exact mass). A combination of exact mass and retention time should be used with caution in the identification of plant food bioactive metabolites because of the high number of existing isomers without guarantee that these do not co-elute in the system used. Figure 4 shows the identification of three flavonoids, cyanidin, luteolin and kaempferol with identical formulae and exact mass (287.0555) present in the same mixture. In this case, unless isolated standards have been analyzed separately in the same platform, only the comparison of MS/MS fragmentation patterns can elucidate the identity of the compounds. With stereoisomers and *cis*–*trans* isomers, the MS/MS spectra often appear too similar to be reliably distinguished (Figure 5). The elution order of *cis* and *trans* isomers depends on the overall structure of the compound and thus cannot be generalized [34]. In this study, the isomers of chlorogenic acid (3-*O*-caffeoylquinic and 5-*O*-caffeoylquinic acid, present as a mixture in the standard), ferulic acid (*cis* isomer produced by UV radiation), and resveratrol (*cis* isomer produced by UV radiation) were chromatographically separated in platforms with varying runtime lengths. A runtime that is too short may hinder the differentiation between such closely eluting compounds, and this should be considered when planning the chromatographic conditions. An alternative is to use a multi-step gradient, as employed in this study for platforms 1, 2, 4, 6, 7, and 9 (Figure 2), which may enhance the separation of certain compounds with similar physicochemical properties; as a drawback, some peak broadening usually occurs.

**Figure 6:**
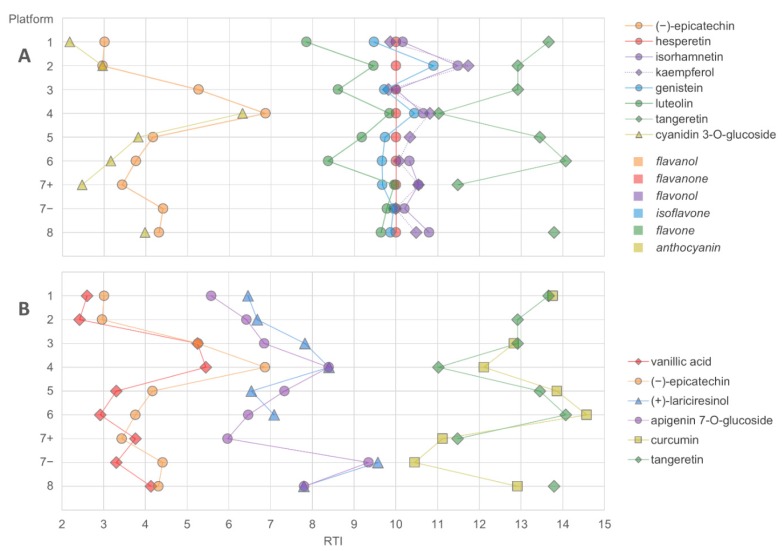
The elution order of flavonoids (**A**) and three pairs of closely eluting compounds representing different compound classes (**B**) in LC–MS platforms using a C_18_ reversed-phase column. The retention times were normalized based on gallic acid and hesperetin (RT_N_ for hesperetin = 10).

The elution order of the studied compounds in the LC–MS platforms was examined by comparing the normalized retention times (gallic acid having RT_N_ = 0 and hesperetin RT_N_ = 10). As shown in Figure 6, the elution order differed across platforms within the same family of compounds (flavonoids) or between closely eluting compounds from different compound classes. In addition, the normalized retention times still varied considerably between the platforms for each compound. This indicates that even if a similar type of column (such as RP C_18_) is used, elution order should be used with caution in annotating compounds, unless the order has been previously validated with standards in the respective chromatographic system. For flavonoids, the elution order has been established at a subclass level for RP-LC as follows: flavanols < flavanones < flavonols < flavones, but differences in selectivity exist, depending on the exact nature of the stationary phase [10]. Based on the results presented here, even the subclass elution order of flavonoids seems to be unpredictable across platforms. The factors contributing to the elution order in HPLC have not been widely studied, but apart from the hydrophobic properties of the analyte (which may be considered the primary indicator of the retentivity), they may include the solvent, acidic modifier and pH of the mobile phase and the physicochemical properties of the column, such as the particle and pore size and the chemistry of the stationary phase. Thus, in untargeted metabolomics analysis, caution and effort should be given for the identification process to ensure that the presented results can be properly supported by the findings.

Because of the nearly infinite options for arranging liquid chromatography, including the choice of column, mobile phase, flow rate and length and shape of the gradient, the elution of analytes will differ in a way that is challenging to predict. In the case of hesperetin, which was detected in all the platforms, the proportion of the organic solvent did not correlate with the retention times (Figure 2). As a lipophilic compound, ursolic acid eluted close to the end of the gradient (during the maximum percentage of solvent B), even in the re-equilibration phase, or did not elute at all. Therefore, lipophilic compounds require a sufficient period of time to be reserved for keeping %B at the highest level. Another issue is related to the solvent within the injected sample: since methanol or similar non-polar solvent is often used in the extraction of metabolites, it can disturb the retention of hydrophilic compounds in the beginning of the chromatographic run. This “falling over” effect can be minimized by reducing the injection volume.

### 2.4. Considerations on the Optimal Coverage of Plant Bioactives

This study revealed the wide diversity of analytical methods used in untargeted metabolomics and the complete lack of standardization so far. Untargeted methods are being developed with the purpose of detecting as many compounds as possible. However, the platforms do not have a precise knowledge of the coverage of their methods until they have analyzed hundreds of standards from all chemical families. In the human nutrition field, in-house spectral libraries always include a wide range of endogenous metabolites, while the metabolites of plant food bioactives are often poorly represented, partly because many standards are expensive or not commercially available. No universal method exists that can cover the diversity of chemical structures present in biological samples. As a discovery-oriented approach, untargeted metabolomics does not necessarily require standardization. Quite different datasets can be obtained from the same samples when analyzed with different methods, and this may lead to complementary findings. However, it is essential for the user to know the coverage and the limitations of the available methods to make appropriate choices according to the study objectives and have correct interpretations of the acquired data.

Some conclusions can be drawn from our results for designing an optimal untargeted analytical method with a wide coverage of plant food bioactives and their metabolites. The choice of instrumentation is the first obvious factor in determining the analytical coverage of analytes, and it has been widely discussed in the literature. Currently, HPLC or UHPLC coupled with qTOF, Orbitrap or ICR mass spectrometer seems to offer widest coverage, as also indicated by the results in the current study. Assuming that the instrument is suitable for metabolomics, there are several other ways to increase the coverage. Regarding the MS part of the method, both positive and negative ionization should be included to increase the coverage and the proportion of identified compounds. MS/MS data should always be acquired for reliable identification.

As for the chromatographic separation, a sufficient but economical liquid chromatography runtime is essential for the separation of closely eluting peaks. The shortest chromatographic runs in the current study performed well for the identification of plant bioactives in solvent, but less well for the urine matrix. In addition, the separation of some closely eluting peaks was weak in some of the platforms, such as with the method used by platform 7, which was developed and validated for targeted quantitative analysis of a large set of dietary metabolites by using the multiple reaction monitoring (MRM) acquisition mode, which allows applying shorter runtimes with high sensitivity and selectivity. For untargeted analysis of complex matrices, a high resolution of peaks is needed and thus, a longer gradient generally performs better. In platforms with longer runtimes (more than 12 min in the normalized gradient, Figure 2B), the coverage increased for the urine matrix and the peaks were well separated. Consequently, we point out that chromatographic separation is an important part of MS-based metabolomics and the direct infusion or “shotgun” approach in MS detection is discouraged when aiming for comprehensive untargeted metabolomics with maximal analytical coverage. This is because, independent of the mass accuracy and mass resolution of the MS instrumentation used, chromatographic separation has an essential contribution in unambiguous identification of closely related isomers such as *cis-trans* epimers. In addition, chromatographic separation is highly advantageous in reducing matrix effects, thus enhancing sensitivity and eventually the metabolite coverage.

Since compounds at the extreme ends of the log *P* scale may be out of reach for a single column, the introduction of another complementary column, such as HILIC, would increase the analytical coverage of highly hydrophilic compounds. Alternatively, sufficient time should be reserved for keeping the percentage of the organic solvent of the mobile phase at its maximum to allow the elution of the most lipophilic compounds. In practice, it seems unrealistic at the present time that platforms that have already put major effort into building large in-house spectral libraries for their own method would easily change it for a standardized recommended one. An alternative is to propose a set of quality controls, based on the analysis of a short selection of standards, which can be used to assess the relevance of the method used regarding plant food bioactive metabolites. Based on the results presented in the current study, we propose certain method development and validation quality control guidelines for acquiring sufficient coverage of plant-based bioactive molecules. The suggested guidelines are as follows:

- The retention capability of the method for highly polar compounds can be tested by compounds such as theobromine and trigonelline. The suitability of the initial solvent composition and injection volume of the HPLC method should be optimized based on the results of these test runs. In an optimal setting, these two hydrophilic compounds (or at least theobromine) should elute after the solvent peak.
- The chromatographic resolution of the method should be challenged by analyzing critical peak pairs. For instance, vanillic acid and (−)-epicatechin are good candidates to test the chromatographic resolution in the initial separation phase. Kaempferol and luteolin are good choices for the mid-polarity range. Stricter evaluation can be performed by using the *cis* and *trans* epimers of ferulic acid or resveratrol.
- The performance of the method for highly non-polar compounds can be tested by ursolic acid and α-tocopherol. These two lipophilic compounds should elute before the solvent gradient with the highest %B (percentage of the organic solvent) reaches the column end.

To perform the above tests and to assess additional method performance attributes such as MS sensitivity in either polarity, we propose here a simple “analytical coverage quality control mixture” as part of the method optimization. It would consist of the following twelve relatively inexpensive and widely available chemical standards used in the current study, covering a wide chemical space:

- α-tocopherol
- chlorogenic acid
- (−)-epicatechin
- ferulic acid
- kaempferol
- luteolin
- naringin
- resveratrol
- theobromine
- trigonelline
- ursolic acid
- vanillic acid

The mixture would cover (i) lipophilic (α-tocopherol, ursolic acid) and hydrophilic (theobromine, trigonelline) compounds, (ii) high molecular weight compounds (naringin), (iii) compounds easily detectable in both positive and negative modes in biological matrices (ferulic acid and luteolin), (iv) closely eluting compounds with identical formulae in the initial phase of chromatography (vanillic acid and (−)-epicatechin) and in the mid-polarity range (kaempferol and luteolin), (v) mixtures of *cis* and *trans* isomers for clear chromatographic separation (ferulic acid and resveratrol), (vi) mixtures of epimers (chlorogenic acid) and (vii) various phytochemical classes. The selected compounds are highlighted in Figure 1. It should be noted that only some of the LC–MS platforms in this study were able to detect all these compounds, thus highlighting the importance of such coverage evaluation.

While very different methods utilizing untargeted metabolomics can yield equally good results, there is clearly a need for a standardized method or at least minimum requirements for sufficient analytical coverage. The development and, most importantly, the implementation of an optimized consensus method would serve the untargeted plant and food metabolomics research by increasing the analytical coverage of plant bioactives in simple and complex matrices, and allowing a more direct comparison of results, including retention times and elution order of compounds. Additionally, existing databases of plant bioactives could include this information in their entries, providing additional confirmation for identification in platforms using the same method. However, practical difficulties may be faced in adopting the use of any single method due to the existing variety of well-performing untargeted metabolomics platforms. Therefore, finding a single consensus method may depend more upon agreements within the scientific community. As future work, using the results from the current study, we plan to design and validate an optimized consensus method and more widely assess the analytical coverage of other bioactive compounds in plant foods, as well as sensitivity and reproducibility of the new method. This method will then be proposed as the consensus method for untargeted LC–MS analyses on plant food bioactives.

## 3. Materials and Methods

### 3.1. Reference Standard Mixtures

The criteria for selecting the chemical standards for the project were (i) to reflect the research interests of the partners involved in the COST POSITIVe network, (ii) to represent a variety of dietary phytochemical classes, and (iii) to include compounds covering a wide range of masses and polarities. An initial list of 181 compounds was established after circulation among POSITIVe partners, based on research interests and previous knowledge of the occurrence of plant bioactives in foods and their metabolism (Table S1 in Supplementary Materials). A second questionnaire was sent to all COST POSITIVe partners to determine the availability of the listed chemicals as pure standards. Considering primarily the coverage of the chemical space and secondarily the price and availability of the standards, the list was narrowed down to 56 representative compounds.

The 56 chemical standards shared by ten POSITIVe partners mostly originated from commercial vendors and 9 compounds had been synthesized in-house. Chemical structures and the origin of the standards are presented in Supplementary Table S2. The log *P* values were calculated for each compound using ALOGPS 2.1 [36]. Based on the log *P* value and literature and database searches (PubChem and ChemSpider), the solubility of the compounds in common solvents was assessed and the standards were dissolved into 10 mM stock solutions with the most appropriate solvent (water:acetonitrile 1:1, methanol, or chloroform). In case of incomplete dissolution or precipitation after visual inspection, the suspension was diluted with the same or a less polar solvent. The final concentration of these compounds, including apigenin 7-*O*-glucoside, bergaptol, catechol-*O*-sulfate, curcumin, ellagic acid, isorhamnetin, luteolin, 5-pentacosylresorcinol, quercetin 3′-*O*-sulfate, quercetin 4′-*O*-sulfate, quercetin disulfate, and sinapic acid, is given in Supplementary Table S2. Ellagic acid was dissolved in methanol into a concentration of 50 ppm (approximately 0.17 mM), as this was the maximum concentration possible to achieve based on previous knowledge. Three standard mixtures, A (water:acetonitrile 1:1 as the solvent), B (methanol), and C (chloroform), were prepared from the stock solutions at 200 µM (or lower in the above-mentioned cases). The presence of the compounds in the mixtures were preliminarily inspected with LC–MS platforms available at the organizing laboratory. A pool of rat urine samples was obtained from the Bioterium at the Faculty of Pharmacy of the University of Lisbon, from animals not being used in any study. The urine samples were collected over a 24-h period from rats placed in a standard commercial metabolism cage with free access to water. The pooled urine aliquots were prepared for the analysis of the standards in the urine matrix.

The three mixtures of chemical standards and the urine samples were sent on dry ice to the participating platforms, along with a SOP (Supplementary Figure S1) describing all the procedures to be followed by the organizing and participating laboratories, as well as instructions and templates for providing the results in a uniform manner.

### 3.2. Sample Preparation and Analysis with Mass Spectrometry

Each participating platform used its routine analytical procedures previously developed for untargeted metabolomics studies. The types of the instruments used and the conditions for chromatographic separation and mass spectrometry are detailed in Table 1. Before the analysis, the mixtures were diluted into a final concentration of 10 µM (with the exceptions in Supplementary Table S1) using water (mixture A) or methanol (mixtures B and C) as a solvent. The 10 µM concentration was chosen to represent the expected concentrations of metabolites in human biofluids, without being too challenging in terms of sensitivity. Each LC–MS platform was asked to report the following results for each detected compound: retention time, ion mode, observed *m*/*z*, mass error in ppm, and signal-to-noise ratio. When needed for discriminating compounds with identical elemental formula, MS/MS fragmentation was performed, and data were reported as the list of the most intense MS/MS fragments with percent relative intensity and collision induced (CID) energy used. In case of discrepancy between the expected and reported *m*/*z*, retention time or MS/MS fragmentation pattern, the platform was contacted for verification.

### 3.3. Data Analysis and Compound Identification

To visually compare data from different platforms with variable chromatographic gradients, retention times from the LC–MS analyses were normalized based on two standards with low RT (gallic acid) and higher RT (hesperetin) using the following formula:

(1) $${\mathrm{RT}}_{N}=10\times \frac{\mathrm{RT}-{\mathrm{RT}}_{\mathrm{gallic}\;\mathrm{acid}}}{{\mathrm{RT}}_{\mathrm{hesperetin}}-{\mathrm{RT}}_{\mathrm{gallic}\;\mathrm{acid}}}$$

Thus, gallic acid was assigned RT_N_ = 0 and hesperetin RT_N_ = 10. For similar comparison and visualization purposes, the chromatographic gradients of the LC–MS platforms were normalized based on a column with dimensions of 2.1 × 100 mm, particle size 1.7 µm, and flow rate 0.4 mL/min using an online HPLC method transfer calculator (Supelco HPLC Calculator for Isocratic or Gradient Method Transfer; Sigma-Aldrich; https://www.sigmaaldrich.com/analytical-chromatography/hplc/method-transfer-calculator.html).

Each platform used their standard method to identify the compounds in the standard mixtures. These methods included using vendor software, Progenesis Qi (platform 2), and MS-DIAL version 2.64 (platform 8), an open-source software for metabolomics data deconvolution and analysis [37].

## Figures and Tables

**Table 1:** Platforms that participated in the test with their equipment information and mass spectrometric and chromatographic conditions. ESI = electrospray ionization, EI = electron ionization, * hydrophilic interaction chromatography (HILIC).

Plat-Form	General Method(s)	HPLC/GC Model	Column (Dimensions, Particle Size, Pore Size)	MS	Ion Source	HPLC Mobile Phase	HPLC Flow	HPLC Gradient (t [min], %B)
1	UHPLC–QTOF	Agilent 1290	Agilent Poroshell 120 EC-C18 (3 × 100 mm, 2.7 µm, 120 Å)	Agilent 6550	ESI+/−	A: H_2_O + 0.1% FA, B: ACN + 0.1% FA	0.4 mL/min	(0, 5), (10, 25), (20, 40), (24, 90), (25, 90), (26, 5), (30, 5)
2	UHPLC–TOF	Acquity H-class	Acquity UPLC BEH Shield RP18 (2.1 × 150 mm, 1.7 µm, 130 Å)	Synapt G2 S	ESI+/−	A: H_2_O + 0.1% FA, B: ACN + 0.1% FA	0.35 mL/min	(0, 5), (30, 50), (31, 100), (37, 100), (37.1, 0)
3	HPLC–QTOF	Thermo U3000	Acquity HSS T3 (2.1 × 150 mm, 1.8 µm, 100 Å)	Bruker Impact HD2	ESI+/−	A: H_2_O + 0.1% FA, B: ACN + 0.1% FA	0.4 mL/min	(0, 0), (2, 0), (15, 100), (22, 100), (22.1, 0), (26, 0)
4	HPLC–QTOF	Thermo U3000	Acquity UPLC BEH Shield RP18 (2.1 × 100 mm, 1.7 µm, 130 Å)	Bruker Impact HD2	ESI+/−	A: H_2_O + 0.1% FA, B: ACN + 0.1% FA	0.4 mL/min	(0, 0), (2, 0), (7, 10), (22, 95), (22.1, 0), (26, 0)
5	UHPLC–QTOF	Eksigent nanoLC	Eksigent HALO C18 (0.5 × 50 mm, 2.7 µm, 90 Å)	Sciex Triple-TOF 6600	ESI+/−	A: H_2_O + 0.1% FA, B: ACN + 0.1% FA	10 µL/min	(0, 5), (12, 95), (14, 95), (16, 5)
6	UHPLC–QTOF	Agilent 1260	Phenomenex Synergi Hydro-RP (2 × 150 mm, 4 µm, 80 Å)	Agilent 6530	ESI+/−	A: H_2_O + 0.1% FA, B: ACN + 0.1% FA	0.5 mL/min	(0, 5), (1, 5), (35, 45), (40, 100), (45, 100)
7+	UHPLC−QTRAP	Agilent 1290	Luna Omega Polar C18 (2.1 × 100 mm, 1.6 µm, 100 Å)	Sciex 6500	ESI+	A: H_2_O + 0.5% FA, B: ACN + 0.5% FA	0.5 mL/min	(0, 5), (5, 50), (5.1, 100), (7, 100), (7.1, 5), (9, 5)
7−	UHPLC−QTRAP	Agilent 1290	Luna Omega Polar C18 (2.1 × 100 mm, 1.6 µm, 100 Å)	Sciex 6500	ESI−	A: H_2_O + 0.1% FA + 10 mM NH_4_COOH, B: ACN	“	(0, 5), (8, 20), (10, 100), (12, 100), (12.1, 5), (14, 5)
8	UHPLC–QTOF	Agilent 1290	Agilent Zorbax Eclipse XDB-C18 (2.1 × 100 mm, 1.8 µm, 80 Å)	Agilent 6540	ESI+/−	A: H_2_O + 0.1% FA, B: MeOH + 0.1% FA	0.4 mL/min	(0, 2), (10, 100), (14.5, 100), (14.51, 2), (16.5, 2)
8H *	UHPLC–QTOF	Agilent 1290	Waters Aqcuity UPLC BEH Amide (2.1 × 100 mm, 1.7 µm, 130 Å)	Agilent 6540	ESI+/−	A: 50% ACN + 20 mM NH_4_COOH, B: 90% ACN + 20 mM NH_4_COOH; pH = 3	0.6 mL/min	(0, 100), (2.5, 100), (10, 0), (10.1, 100), (14, 100)
9	HPLC–QTOF	Agilent 1200	Luna C18 (4.6 × 150 mm, 3 µm, 100 Å)	Agilent G6530A	ESI+/−	A: H_2_O + 0.1% FA, B: ACN + 0.1% FA	0.5 mL/min	(0, 0), (30, 30), (35, 40), (50, 80), (52, 80), (60, 0), (65, 0)
10	HPLC–QTOF	AB Sciex MicroLC 200	Eksigent HALO C18 (0.5 × 100 mm, 2.7 µm, 90 Å)	AB Sciex 5600+	ESI+/−	A: H_2_O + 0.9% FA, B: ACN + 0.9% FA	15 µL/min	(0, 0), (0.5, 0), (4, 95), (4.5, 1), (5, 1)
11	HPLC–MS	Shimadzu Prominence	Kinetex PFP (4.6 × 150 mm, 5 µm, 100 Å)	Shimadzu LCMS 2020	ESI+/–	A: H_2_O + 0.1% TFAB: MeOH	0.4 mL/min	(0, 40), (25, 80)
12	GC–MS	Shimadzu 2010 Plus	Restek Rxi-5 ms (15 m, 0.25 mm, 0.25 µm)	Shimadzu TQ-8030	EI	-	-	-
13	GC–MS	Agilent 7890A	DB-5 ms (30 m, 0.25 mm, 0.25 µm)	Agilent 5975C MSD	EI	-	-	-

## Supplementary Materials

The following are available online at http://www.mdpi.com/2218-1989/8/3/46/s1, Figure S1: The contents of the standard operating procedure (SOP) sent to each participating platform, Table S1: A list of 181 plant bioactives and their metabolites selected as candidates for the multiplatform analysis classified based on their chemical or metabolite class, Table S2: Chemical standards analyzed in the multiplatform test.
