# Supplementary material for: Interlaboratory Coverage Test on Plant Food Bioactive Compounds and Their Metabolites by Mass Spectrometry-Based Untargeted Metabolomics
Source: Metabolites. 2018 Aug 24;8(3):46. doi: 10.3390/metabo8030046 (PMC6161174; doi:10.3390/metabo8030046)
Supplement: Supplementary file 1 [file metabolites-08-00046-s001.pdf]

Supplementary material

Supplementary Table 1. A list of 181 plant bioactives and their metabolites selected as candidates for the multiplatform analysis classified based on their chemical or metabolite class.

**Carotenoids**
Lutein
Lycopene
Zeaxanthin
β-Carotene
β-Cryptoxanthin
**Phytosterols**
Brassicasterol
Campesterol
Stigmasterol
β-Sitosterol
**Phenolic acids**
2-Hydroxycinnamic acid
5-Feruloylquinic acid
Caffeic acid
Chlorogenic acid
p-Coumaric acid
Ellagic acid
Ferulic acid
Gallic acid
Homovanillic acid
Protocatechuic acid
Rosmarinic acid
Sinapic acid
Vanillic acid
Veratric acid
Verbascoside
**Flavonoids**
(−)-Epicatechin
(−)-Epigallocatechin 3-*O*-gallate
8-Prenylnaringenin
Apigenin
Apigenin 7-*O*-glucoside
Cyanidin
Cyanidin 3-*O*-glucoside
Cyanidin 3-*O*-rutinoside
Daidzein
Delphinidin
Dihydrogenistein

Diosmetin
Eriodictyol
Genistein
Hesperetin
Homoeriodictyol
Homoorientin
Isoliquiritigenin
Isoquercitrin
Isorhamnetin
Isosakuranetin
Kaempferol
Luteolin
Malvidin 3-*O*-glucoside
Myricetin
Naringenin
Naringin
Nobiletin
Phloretin
Procyanidin A2
Procyanidin B1
Procyanidin B2
Procyanidin B4
Quercetin
Silybin
Sinensetin
Tangeretin
Taxifolin
Vitexin
Xanthohumol

Other polyphenols

(+)-lariciresinol
5-heptadecylresorcinol (AR17:0)
5-pentacosylresorcinol (AR25:0)
α-Tocopherol
Curcumin
Hydroxytyrosol
Resveratrol
Vanillin

Glucuronides

3'-Methylcyanidin 3-glucuronide
4-Hydroxybenzoic acid 4-*O*-glucuronide
Apigenin 7-glucuronide
Caffeic acid 3-*O*-β-D-glucuronide
cis-Resveratrol 3-*O*-β-D-glucuronide
cis-Resveratrol 4'-*O*-β-D-glucuronide
Curcumin 4-*O*-β-D-glucuronide

Cyanidin 3-glucuronide
Daidzein 4’-β-D-glucuronide
Daidzein 7-β-D-glucuronide
Daidzein diglucuronide
Diosmetin 3'-*O*-β-D-glucuronide
Epicatechin 3-*O*-glucuronide
Genistein 4’-β-D-glucuronide
Genistein 7-β-D-glucuronide
Genistein diglucuronide
Hesperetin 3'-*O*-β-D-glucuronide
Hesperetin 7,3'-di-*O*-β-D-glucuronide
Hesperetin 7-*O*-β-D-glucuronide
Isorhamnetin 3-*O*-glucuronide
Isorhamnetin 4'-*O*-glucuronide
Isovanillic acid 3-*O*-glucuronide
Kaempferol 3-*O*-β-D-glucuronide
Luteolin 7-*O*-β-D-glucuronide
Myricetin 3'-*O*-glucuronide
Naringenin 4'-*O*-β-D-glucuronide
Naringenin 7-*O*-β-D-glucuronide
Protocatechuic acid 4-*O*-glucuronide
Quercetin 3'-*O*-β-D-glucuronide
Quercetin 5,7-diglucuronide
Quercetin 7-*O*-β-D-glucuronide
trans-Resveratrol 3-*O*-β-D-glucuronide
trans-Resveratrol 4'-*O*-β-D-glucuronide
Vanillic acid 4-*O*-glucuronide

Sulfates

1-Methylpyrogallol 3-O-sulfate
2-Methylpyrogallol 1-O-sulfate
3,4-Dihydroxybenzoic acid 3-*O*-sulfate
3,4-Dihydroxybenzoic acid 4-*O*-sulfate
3'-O-Methyl-(-)-epicatechin 5-*O*-sulfate
3'-O-Methyl-(-)-epicatechin 7-*O*-sulfate
4-Hydroxybenzoic acid 4-O-sulfate
4-Methylcatechol 1-O-sulfate
4-Methylcatechol 2-O-sulfate
4-O-Methylgallic acid 3-O-sulfate
Benzoic acid sulfate
Caffeic acid 3-O-sulfate
Caffeic acid 4-O-sulfate
Catechol *O*-sulfate
Daidzein 4' sulfate
Epicatechin 3-*O*-sulfate
Genistein 7-sulfate
Isoferulic acid 3-*O*-sulfate

Isoquercitrin 4'-*O*-sulfate
Isorhamnetin 3-*O*-sulfate
Isovanillic acid 3-*O*-sulfate
Kaempferol 3-*O*-sulfate
Myricetin 3'-*O*-sulfate
Protocatechuic acid 3-O-sulfate
Protocatechuic acid 4-O-sulfate
Pyrogallol 2-*O*-sulfate
Quercetin 3'-*O*-sulfate
Quercetin 4'-*O*-sulfate
Quercetin disulfate
Silybin 20-*O*-sulfate
Silybin 7,20-di-*O*-sulfate
Taxifolin 4'-*O*-sulfate
Vanillic acid 4-*O*-sulfate

Sulfates and glucuronides

3'-Methylcyanidin 3-glucuronide-5-glucoside-4'-sulfate
Daidzein 7-β-D-glucuronide 4’-Sulfate
Genistein 7-sulfate 4’-β-D-glucuronide
Genistein 7-β-D-glucuronide 4’-sulfate

Microbial metabolites

3,4-Dihydroxybenzoic acid
3,4-Dihydroxyphenylacetic acid
3,4-Dihydroxyphenylpropionic acid
3,4-Dimethoxybenzoic acid
3-Hydroxybenzoic acid
3-Hydroxyphenylacetic acid
3-Hydroxyphenylpropionic acid
3-Phenylpropionic acid
4-Coumaric acid
4-Hydroxybenzoic acid
4-Hydroxyphenylacetic acid
4-Hydroxyphenylpropionic acid
4-Methylcatechol
Benzoic acid
Dihydrocaffeic acid
Dihydrodaidzein
Enterodiol
Enterolactone
Equol
Equol 4'-sulfate
Equol 7-β-D-glucuronide
Hippuric acid
O-Desmethylangolensin
Protocatechuic acid

Urolithin A
Urolithin A 3-glucuronide
Urolithin A 8-glucuronide
Urolithin B
Urolithin B 3-*O*-glucuronide
Urolithin C
Urolithin D

Other compounds

Bergaptol
Cafestol
myo-Inositol
Stachydrine
Theobromine
Trigonelline
Ursolic acid

Supplementary Table 2. Chemical standards analysed in the multiplatform test. Concentration in the stock solution other than 10 mM: * 5 mM, † 1 mM, ‡ 0.5 mM, § 50 ppm

Compound	PhytoHub ID	Class	Formula	M_0 [Da]	log *P* (calc.)	Supplier	Mix	Structure
trigonelline	PHUB001378	betaine	$C_7H_7NO_2$	137.0477	−3.30	Extrasynthese	A	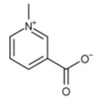
stachydrine (proline betaine)	PHUB000844	betaine	$C_7H_{13}NO_2$	143.0946	−2.24	Extrasynthese	A	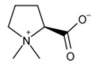
4-hydroxyphenylacetic acid	PHUB000543	microbial metabolite	$C_8H_8O_3$	152.0473	0.93	Aldrich	A	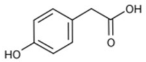
vanillin	PHUB000645	other phenolic	$C_8H_8O_3$	152.0473	1.31	Sigma-Aldrich	A	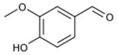
protocatechuic acid	PHUB000310	phenolic acid	$C_7H_6O_4$	154.0266	1.32	Sigma	A	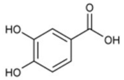
hydroxytyrosol	PHUB001321	other phenolic	$C_8H_{10}O_3$	154.0630	0.13	Extrasynthese	B	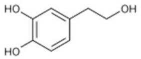
p-coumaric acid	PHUB000590	phenolic acid	$C_9H_8O_3$	164.0473	1.74	Sigma	A	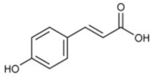
3-(4-hydroxyphenyl)-propionic acid	PHUB001072	microbial metabolite	$C_9H_{10}O_3$	166.0630	1.15	Fluka	A	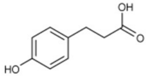
vanillic acid	PHUB000316	phenolic acid	$C_8H_8O_4$	168.0423	1.70	Sigma	A	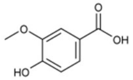
gallic acid	PHUB000303	phenolic acid	$C_7H_6O_5$	170.0215	1.17	Sigma (G73849	A	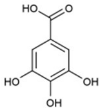

hippuric acid	PHUB001174	phenolic acid	$C_9H_9NO_3$	179.0582	0.23	Sigma (112003)	A	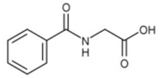
caffeic acid	PHUB000574	phenolic acid	$C_9H_8O_4$	180.0423	1.67	Sigma (C0625)	A	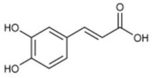
myo-inositol	PHUB001870	sugar alcohol	$C_6H_{12}O_6$	180.0634	−2.59	Merck	A	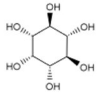
theobromine	PHUB000790	alkaloid	$C_7H_8N_4O_2$	180.0650	−0.46	Extrasynthese	A	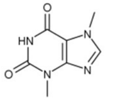
dihydrocaffeic acid	PHUB000604	microbial metabolite	$C_9H_{10}O_4$	182.0579	1.04	Sigma	A	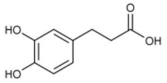
homovanillic acid	PHUB000617	phenolic acid	$C_9H_{10}O_4$	182.0579	1.02	Extrasynthese	B	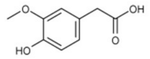
veratric acid	PHUB001218	phenolic acid	$C_9H_{10}O_4$	182.0579	1.52	Aldrich	A	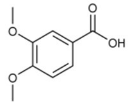
catechol *O*-sulfate	PHUB001352	sulfate	$C_6H_6O_5S$	189.9936	−0.81	*synthesised in-house*	B[†]	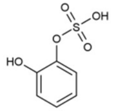
ferulic acid	PHUB000608	phenolic acid	$C_{10}H_{10}O_4$	194.0579	1.58	Aldrich (128708)	A	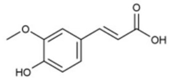
bergaptol	PHUB000259	other phenolic	$C_{11}H_6O_4$	202.0270	1.86	Extrasynthese	B*	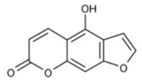
pyrogallol 2-*O*-sulfate	PHUB001416	sulfate	$C_6H_6O_6S$	205.9885	−0.66	*synthesised in-house*	A	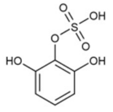

urolithin B	PHUB001394	microbial metabolite	$C_{13}H_8O_3$	212.0473	2.65	Villapharma Research S.L.	B	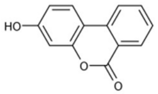
sinapic acid	PHUB000638	phenolic acid	$C_{11}H_{12}O_5$	224.0685	1.63	Sigma	A†	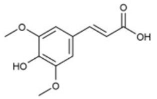
urolithin A	PHUB001391	microbial metabolite	$C_{13}H_8O_4$	228.0423	2.16	Villapharma Research S.L.	B	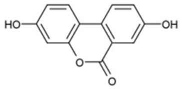
resveratrol	PHUB000324	other phenolic	$C_{14}H_{12}O_3$	228.0786	2.57	Sigma	A	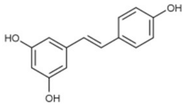
4-*O*-methylgallic acid 3-*O*-sulfate	PHUB001873	sulfate	$C_8H_8O_8S$	263.9940	−0.53	*synthesised in-house*	A	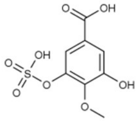
genistein	PHUB000225	flavonoid	$C_{15}H_{10}O_5$	270.0528	3.04	Extrasynthese	B	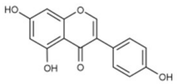
phloretin	PHUB001344	other phenolic	$C_{15}H_{14}O_5$	274.0841	2.23	Extrasynthese	B	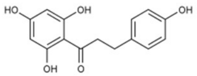
kaempferol	PHUB000672	flavonoid	$C_{15}H_{10}O_6$	286.0477	1.99	Extrasynthese	B	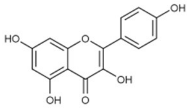
luteolin	PHUB000892	flavonoid	$C_{15}H_{10}O_6$	286.0477	2.73	Fluka	B*	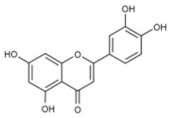
cyanidin	PHUB000483	flavonoid	$C_{15}H_{11}O_6^+$	287.0556	2.41	n/a	B	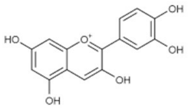

(−)-epicatechin	PHUB000262	flavonoid	$C_{15}H_{14}O_6$	290.0790	1.02	Sigma	B	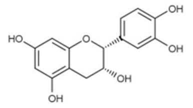
ellagic acid	PHUB000298	phenolic acid	$C_{14}H_6O_8$	302.0063	1.59	Sigma	A[§]	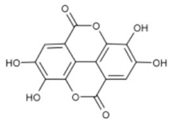
hesperetin	PHUB000380	flavonoid	$C_{16}H_{14}O_6$	302.0790	2.52	Sigma	A	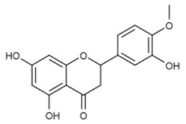
isorhamnetin	PHUB000662	flavonoid	$C_{16}H_{12}O_7$	316.0583	1.96	Extrasynthese	B[‡]	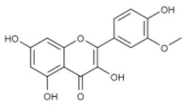
cafestol	PHUB000006	terpenoid	$C_{20}H_{28}O_3$	316.2038	3.04	MP Biomedicals	A	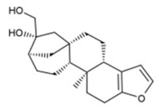
5-heptadecylresorcinol (AR17:0)	PHUB000554	alkylresorcinol	$C_{23}H_{40}O_2$	348.3028	8.79	ReseaChem	B	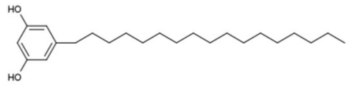
chlorogenic acid	PHUB000585	phenolic acid	$C_{16}H_{18}O_9$	354.0950	0.17	Aldrich (C3878)	A	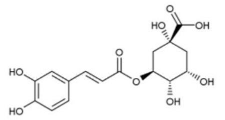
rosmarinic acid	PHUB000634	phenolic acid	$C_{18}H_{16}O_8$	360.0845	2.57	Extrasynthese	A	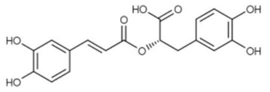
(+)-lariciresinol	PHUB001390	lignan	$C_{20}H_{24}O_6$	360.1573	2.16	ArboNova	A	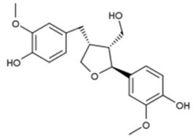
curcumin	PHUB001408	other phenolic	$C_{21}H_{20}O_6$	368.1260	3.62	Sigma	B*	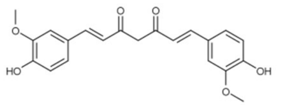

tangeretin	PHUB000907	flavonoid	$C_{20}H_{20}O_7$	372.1210	2.88	Extrasynthese	B	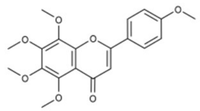
quercetin 3'-*O*-sulfate	PHUB001316	sulfate	$C_{15}H_{10}O_{10}S$	381.9995	0.65	*synthesised in-house*[29]	A†	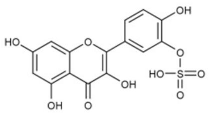
quercetin 4'-*O*-sulfate	PHUB001871	sulfate	$C_{15}H_{10}O_{10}S$	381.9995	0.70	*synthesised in-house*[29]	A†	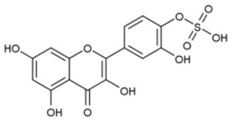
β-sitosterol	PHUB000479	steroid	$C_{29}H_{50}O$	414.3862	7.27	Sigma	C	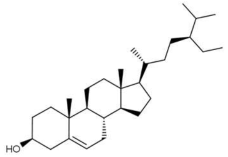
α-tocopherol	PHUB001872	other phenolic	$C_{29}H_{50}O_2$	430.3811	8.84	Sigma	A	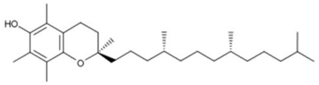
apigenin 7-*O*-glucoside	PHUB000865	flavonoid	$C_{21}H_{20}O_{10}$	432.1056	0.68	HWI Analytik	B*	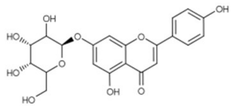
cyanidin 3-*O*-glucoside	PHUB000503	flavonoid	$C_{21}H_{21}O_{11}^{+}$	448.1006	0.98	Extrasynthese	B	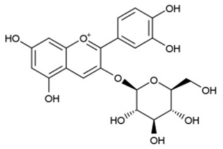
ursolic acid	PHUB000190	terpenoid	$C_{30}H_{48}O_3$	456.3603	6.35	Aldrich	A	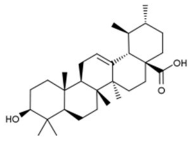
5-pentacosylresorcinol (AR25:0)	PHUB000560	alkylresorcinol	$C_{31}H_{56}O_2$	460.4280	10.4	ReseaChem	B*	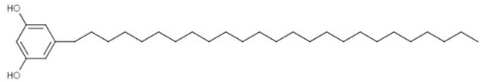

quercetin disulfate	PHUB001874	sulfate	$C_{15}H_{10}O_{13}S_2$	461.9563	−0.34	*a mixture of isomers synthesised in-house*[29]	B[‡]	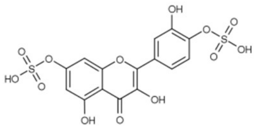
kaempferol 3-glucuronide	PHUB001318	glucuronide	$C_{21}H_{18}O_{12}$	462.0798	1.40	Extrasynthese	A	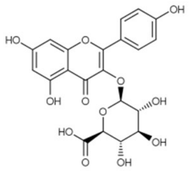
β-carotene	PHUB000350	terpenoid	$C_{40}H_{56}$	536.4382	9.72	Extrasynthese	C	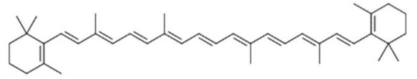
procyanidin A2	PHUB000275	flavonoid	$C_{30}H_{24}O_{12}$	576.1268	2.43	Extrasynthese	A	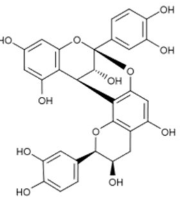
naringin	PHUB000389	flavonoid	$C_{27}H_{32}O_{14}$	580.1792	−0.24	Sigma	A	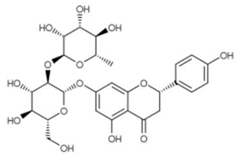
verbascoside	PHUB000646	phenolic acid	$C_{29}H_{36}O_{15}$	624.2054	1.09	Extrasynthese	A	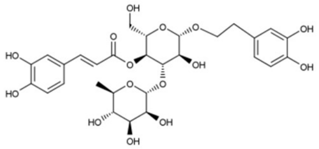

POSITIVe	COST Action FA 1403<br>Interindividual variation in response to consumption of plant food bioactives and determinants involved (POSITIVe)	cost

Contents

1. AIM 3

2. DOCUMENTS GENERATED BY THIS SOP 3

3. PROCEDURE 4

3.1. Preparation of stock solutions 4

3.1.1. Compounds to include in the coverage test 4

3.1.2. Collection of compounds to include in the ring test 4

3.1.3. Preparation of the individual standard stock solutions at 10mM 4

3.1.4. Preparation of the standard mix solution at 200µM (S1) 4

3.1.5. Preparation of the standard stock solution at 10 µM (S2 and U2) 5

3.2. Analysis of standard solutions S2 and U2 5

3.3. Treatment of results 6

Annex 1. List of Analytical platforms envolved and contact persons 7

Annex 2. List of compounds, formula, monoisotopic mass (MM), log P, origin, purity, solubility 8

Annex 3. Characteristics of the MS equipment and conditions of analysis 9

Annex 4. Characteristics of the GC-MS equipment and conditions of analysis 12

Annex 5. Characteristics of the NMR equipment and conditions of analysis 13

Annex 6. Template to provide results from each platform 14

Supplementary Figure 1. The contents of the standard operating procedure (SOP) sent to each participating platform.
